# Synthesis and Biological Activity of 2′,3′-*iso*-Aryl-abscisic Acid Analogs

**DOI:** 10.3390/molecules22122229

**Published:** 2017-12-15

**Authors:** Chuan Wan, Mingan Wang, Dongyan Yang, Xiaoqiang Han, Chuanliang Che, Shanshan Ding, Yumei Xiao, Zhaohai Qin

**Affiliations:** 1 College of Science, China Agricultural University, Beijing 100193, China; wanchuan@cau.edu.cn (C.W.); wangma@cau.edu.cn (M.W.); yangdy@cau.edu.cn (D.Y.); chechuanliang@cau.edu.cn (C.C.); dss_5233@163.com (S.D.); xiaoyumei@cau.edu.cn (Y.X.); 2 College of Agriculture, Shihezi University, Shihezi 832000, China; hancau@cau.edu.cn

**Keywords:** abscisic acid, *iso*-benzoabscisic acid (*iso*-PhABA), *iso*-PhABA analogs, ABA like activities

## Abstract

2′,3′-*iso*-Benzoabscisic acid (*iso*-PhABA), an excellent selective abscisic acid (ABA) analog, was developed in our previous work. In order to find its more structure-activity information, some structural modifications were completed in this paper, including the substitution of phenyl ring and replacing the ring with heterocycles. Thus, 16 novel analogs of *iso*-PhABA were synthesized and screened with three bioassays, Arabidopsis and lettuce seed germination and rice seedling elongation. Some of them, i.e., 2′,3′-*iso*-pyridoabscisic acid (*iso*-PyABA) and 2′,3′-*iso*-franoabscisic acid (*iso*-FrABA), displayed good bioactivities that closed to *iso*-PhABA and natural (+)-ABA. Some others, for instance, substituted-*iso*-PhABA, exhibited certain selectivity to different physiological process when compared to *iso*-PhABA or (+)-ABA. These analogs not only provided new candidates of ABA-like synthetic plant growth regulators (PGRs) for practical application, but also new potential selective agonist/antagonist for probing the specific function of ABA receptors.

## 1. Introduction

**Figure 1:**
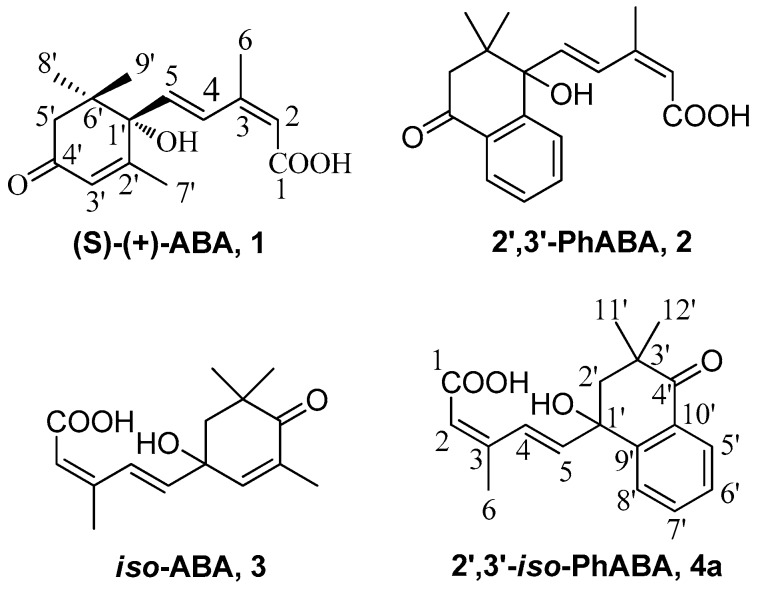
Abscisic acid (ABA) and ABA analogs.

The crucial role of abscisic acid (*S*-(+)-ABA **1**, Figure 1) as a phytohormone in a wide variety of physiological processes of plants, including inhibiting or promoting growth, maintaining bud and seed dormancy, affecting flowering and sex differentiation, as well as conducting environmental stresses response [1,2,3,4,5,6,7,8], motivates numerous scientific investigations into the agricultural application of ABA. However, the widely using of abscisic acid (ABA) has been hindered by its rapid metabolism in plants and light isomerization in vitro [9,10,11,12,13,14,15,16,17]. Focusing on the development of metabolism resistant analogs, significant efforts were devoted by different researchers. For example, methoxyl [18], alkynyl [19], or fluorine atoms [20,21] at the 8′ or 9′-positionm and halogen atoms [22,23] at the 3′-position had been introduced to avoid 8′-hydroxylation and Michael addition, and the corresponding analogs displayed excellent ABA-like activities. Nonetheless, they are too expensive to practical use in field due to their complicated synthetic routes.

**Figure 2:**
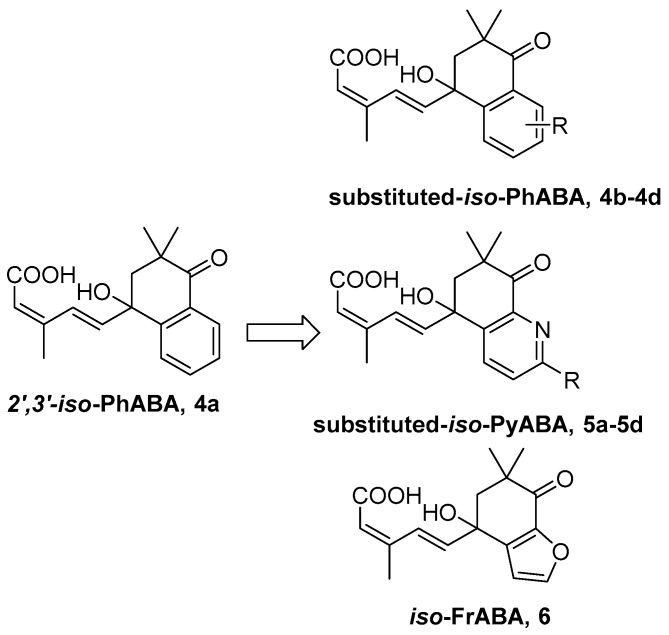
Design strategy of 2′,3′-*iso*-Benzoabscisic acid (*iso*-PhABA) **4a** analogs.

A successful case of 8′-hydroxylation resistant analog was 2′,3′-benzoabscisic acid **2** [24,25] (Figure 1), which had excellent bioactivity and relatively efficient synthetic route. Inspired by this compound and *iso*-ABA **3** [26], in our previous works, we had developed 2′,3′-*iso*-Benzoabscisic acid (*iso*-PhABA) **4a** [27], which is an excellent and easier preparation ABA analog. The deeply investigations of bioactivity and agonist-receptor interactions for **4a** suggested that it is a state-of-art ABA-like regulator and a selective ABA agonist, i.e., PYL10 has the highest inhibitory effect on the phosphatase activity of HAB1 in the presence of (+)-*iso*-PhABA. Meanwhile, the analysis on the complex crystal structure of *iso*-PhABA with PYL10 revealed the detailed hydrogen bonds and multiple hydrophobic interactions. Thus, it provides a new and robust precursor for the design of ABA receptor agonists/antagonists. Consequently, in this work, we aimed at the further studies of the analogs of 2′,3′-*iso*-PhABA **4a** to obtain more compounds with good ABA-like bioactivity and selective agonist/antagonists-receptor interactions. Here, we reported the synthesis and biological activity of substituted-*iso*-PhABA analogs **4b**–**4d** and heterocyclic analogs **5** and **6** (see Figure 2). Their structure-activity relationship (SAR) was also discussed.

## 2. Results and Discussion

### 2.1. Chemistry

**Scheme 1:**
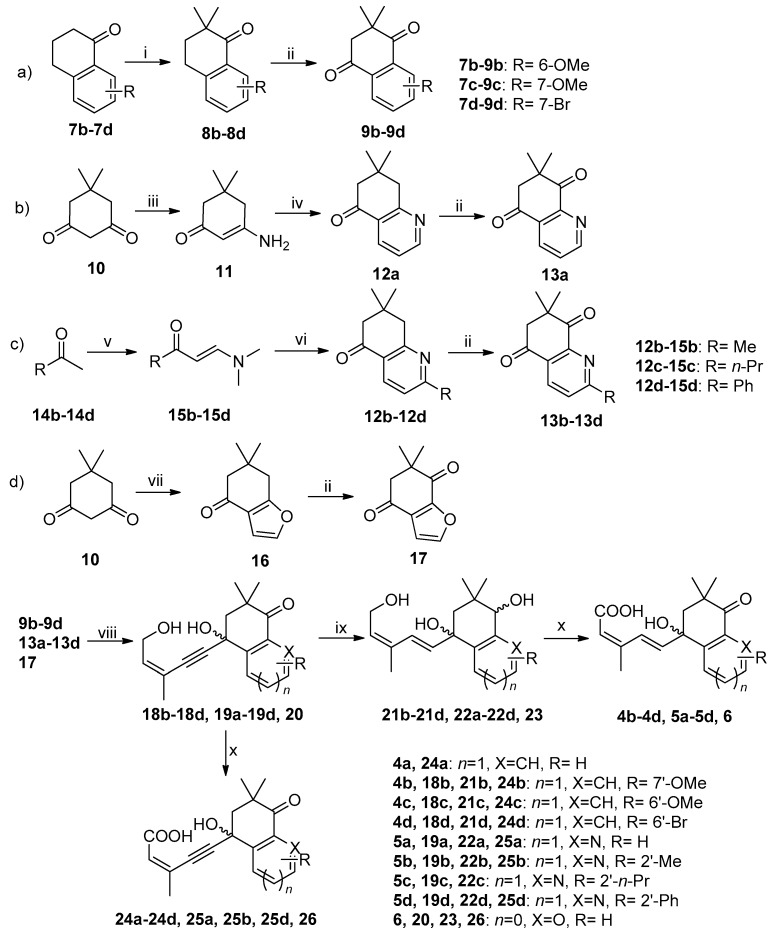
Synthesis of substitueted-*iso*-PhABA analogs **5** and heterocyclic analogs substituted-*iso*-HetABA **6**. Reagents and conditions: (i) MeI, NaH, THF; (ii) Co(acac)_2_, *t*-BuOOH, acetone, r.t.; (iii) NH_4_OAc, toluene, reflux; (iv) 1,1,3,3-tetraethoxylpropane, p-toluenesulfonic acid, DMF, reflux; (v) dimethylformamide dimethylacetal, xylene, reflux; (vi) 5,5-dimethylcyclohexane-1,3-dione, HOAc, reflux; (vii) ClCH_2_CHO, KOH, KI, H_2_O, r.t.; (viii) *n*-BuLi, (*Z*)-3-methylpent-2-en-4-yn-1-ol, THF, −78 °C; (ix) Red-Al reagent, THF, 0 °C; x. (**a**) Dess-Martin periodinane, CH_2_Cl_2_, r.t.; (**b**) NaClO_2_, 2-methyl-2-butene, NaH_2_PO_4_, *t*-BuOH/H_2_O (3:1 in volume), r.t.

As shown in Scheme 1, to obtain target compounds substituted-*iso*-PhABA (**4b**–**4d**) and heterocyclic analogs 2′,3′-*iso*-heterocylicABA (*iso*-HetABA, **5a**–**5d**, **6**), four different pathways (a–d in Scheme 1) for the preparation of dicarbonyl intermediates **9b**–**9d**, **13a**–**13d**, and **17** were established, respectively. Firstly, the preparation of intermediates **9b**–**9d** (Scheme 1a) were followed the similar pathway of the synthesis of 2′,3′-*iso*-PhABA **4** [25,27], comprising the vicinal methylation of the carbonyl of subtituted-1-tetralones (step i in Scheme 1a) to obtain **8b**–**8d** and the oxidation of the benzyl carbon with Co(acac)_2_/*t*-BuOOH system [28] (step ii in Scheme 1a). Then, for the preparations of pyridine analogs **13a**–**13d**, two different methods were applied for the intermediates **12a**–**12d**. The reaction of 5,5-dimethylcyclohexane-1,3-dione with ammonium acetate afforded **11** (step iii in Scheme 1b), and then intermediate **11** reacted with commercially available 1,1,3,3-tetraethoxylpropane and catalytic amount of *p*-toluenesulfonic acid to afford **12a** (step iv in Scheme 1b), which can oxide by Co(acac)_2_/*t*-BuOOH system to afford the dione **13a** (step ii in Scheme 1b) [29,30]. The preparation of the diones **13b**–**13d**, on the other hand, were conducted by three steps, including the condensation of methyl ketones with dimethylformamide dimethylacetal to afford **15b**–**15d** (step v in Scheme 1c), the pyridine ring closure between enamino ketones and 5,5-dimethylcyclohexane-1,3-dione in refluxing acetic acid to afford **12b**–**12d** (step vi in Scheme 1c), and the oxidation to afford diones **13b**–**13d** (step ii in Scheme 1c) [31]. Moreover, 2 steps, comprising the furan ring closure between 5,5-dimethylcyclohexane-1,3-dione and ClCH_2_CHO to afford **16** (step vii in Scheme 1d) and the oxidation to afford dione **17** (step ii in Scheme 1d), were applied for the preparation of furan analog **6** [32,33].

With the diones in hands, the synthesis of the title compounds, i.e., substituted-*iso*-PhABA (**4b**–**4d**) and *iso*-HetABA analogs (**5a**–**5d**, **6**), were performed following an efficient region-selective nucleophilic addition of alkynyl lithium, which was produced in situ by *n*-BuLi and (*Z*)-3-methylpent-2-en-4-yn-1-ol, to the less-steric 4-carbonyl of intermediates **18b**–**18d**, **19a**–**19d**, and **20** (step viii in Scheme 1) [34]. Then, the Red-Al was employed for the *trans*-selective reduction of alkynol to give enol **21b**–**21d**, **22a**–**22d**, and **23** (step ix in Scheme 1). The final products **4b**–**4d**, **5a**–**5d**, and **6** were yielded by a two steps oxidation (step x in Scheme 1), comprising Dess-Martin oxidation to form the aldehydes and then Lindgren oxidation to give the acids **5** and **6**. Furthermore, the alkynol intermediates **18**–**20** were also followed the two steps oxidation to give the acetylenic acid analogs **24b**–**24d**, **25a**, **25b**, **25d**, and **26** (except 2′-*n*-Pr-*iso*-PyABA), since they are good materials for the screening of structural requirement of the trans-double bond of ABA analogs. Meanwhile, the acetylenic *iso*-PhABA **24a** was synthesized following the similar pathway of **24b**–**24d**, with the known intermediate (*Z*)-(1′-hydroxy-3′,3′-dimethyl-4′-oxo-tetrahydronaphthalene-one-yl)-3-methyl-pentyl-2-em-4-yn-1-ol [27].

### 2.2. Structure-Activity Relationship

The *Arabidopsis*, lettuce seed germination and the rice seedling elongation inhibiting tests in vivo were conducted to gain insights into the bioactivity of these *iso*-PhABA analogs. The (±)-*iso*-PhABA **4a** and (+)-ABA **1** were used as the control agents. The corresponding data of bioactivities were summarized in Table 1. For the comparison between the bioactivities of substituted-*iso*-PhABA analogs, the overall bioactivity of **4b**–**4d** were very close to those of each other, indicating that the effects of electron-donating and withdrawing groups, as well as their substituted-positions (7′ and 6′) were not crucial for their bioactivity. However, the bioactivities of all the substituted-*iso*-PhABA **4b**–**4d** were worse than those of the control agents **4a** and **1**, suggesting that a substituent in the benzene ring of *iso*-PhABA had a significant impact on the bioactivity. Furthermore, there were some preferences of substituted-*iso*-PhABA to different physiologic process. Compound **4b**–**4d** observed the highest IC_50_ values for the rice seedlings elongation, which was the lowest values for both the two control agents **4a** and **1**, indicating their different preferential nature of bioactivity compared to *iso*-PhABA.

For the bioactivity of the heterocyclic analogs *iso*-HetABA, the compounds without substituent, i.e*.*, 2′,3′-*iso*-pyridoabscisic acid (*iso*-PyABA) **5a** and 2′,3′-*iso*-franoabscisic acid (*iso*-FrABA) **6**, had good overall bioactivities, which were close to the control agents **4a** and **1**, suggesting the introducing of heteroatom (N for **5a** and O for **6**) in the aryl ring of *iso*-PhABA can mostly keep the bioactivity. To study the effect of the size of the substituted group on the bioactivity, compound **5b**–**5d** were designed and synthesized for the bioassays, too. Obviously, the IC_50_ values of these compounds are decreasing with the increasing size of the substituted, i.e*.*, –Me **5b** to –*n*-Pr **5c** to –Ph **5d**. Therefore, this evidence validated that a big group in the aryl ring is not preferential for the bioactivity.

In order to acquire more analogs for the discussion of structure-activity relationship, the acetylenic acids **24**, **25**, and **26** were also synthesized and tested with three bioassays. As shown in Table 1, their bioactivities were significantly worse than those of their corresponding alkene acids **4**, **5**, and **6**, suggesting the importance of the double-bond for the bioactivity. Besides, by the comparison between the acetylenic acids, acetylenic *iso*-PhABA **24a** had the best activity for *Arabidopsis* seed germination (IC_50_ = 1.38 μM), acetylenic 2′-Me-*iso*-PyABA **25b** had the best activity for lettuce seed germination (IC_50_ = 5.97 μM), and acetylenic *iso*-FrABA **26** had the lowest IC_50_ value (4.32 μM) for rice seedlings elongation. Meanwhile, like the substituted-*iso*-PhABA analogs, there were obvious preferences of some acetylenic acids to different physiological process. For example, acetylenic 7′-OMe-*iso*-PhABA **24b** showed a weakest inhibitor on *Arabidopsis* seed germination (IC_50_ > 10 μM) in three bioassays; acetylenic 6′-Br-*iso*-PhABA **24d**, on the other hand, had a lowest bioactivity on rice seedlings elongation (IC_50_ > 10 μM). Hence, these results implied that there might be some difference in ABA signaling transduction between different acetylenic acids, as well as the acetylenic acids and alkene acids.

## 3. Materials and Methods

### 3.1. Materials and Instruments

Column chromatography was performed using silica gel (100–200 mesh) (Qindao Haiyang Co., Ltd., Qingdao, China). TLC was performed on GF254 silica gel plates (Qingdao Haiyang Co., Ltd., Qingdao, China). NMR spectra were recorded on a Bruker Avance DPX300 spectrometer (Bruker Corporation, Vienna, Austria) with TMS as the internal standard. HR-MS data were obtained on a Thermo FisherScientific LTQ Orbitrap (Thermo Fisher Scientific, Bremen, Germany) instrcument.

### 3.2. Synthesis

#### 3.2.1. Synthesis of **9b**–**9d** (Pathway a in Scheme 1, Use **9b** as Example)

6-Methoxy-2,2-dimethyl-3,4-dihydronaphthalen-1(2*H*)-one (**8b**) [25,27]: To a stirred solution of 6-methoxy-3,4-dihydronaphthalen-1(2*H*)-one **7b** (10.0 g, 56.8 mmol) dissolved in dry tetrahydrofuran (THF, 150 mL) in a 500 mL round bottomed flask was added NaH (11.4 g, 284 mmol, 60% in oil). After stirring the mixture for 1 h under ice-water bath, methyl iodide (32.3 g, 227 mmol) in dry THF (50 mL) was added slowly. The mixture was allowed to stir at r.t. for 16 h. The reaction was quenched by addition of water (slowly and dropwise). The mixture was then extracted with ethyl acetate (3 × 100 mL), washed with water (2 × 100 mL), and dried over anhydrous Na_2_SO_4_. Evaporation of the solvent yielded a yellow oil. The residue was subjected to silica gel chromatography using petroleum ether (PE) and ethyl acetate (EtOAc) (10:1) as eluent to afford 6-methoxy-2,2-dimethyl-3,4-dihydronaphthalen-1(2*H*)-one **8b** (10.4 g, yield 90%) as a yellow oil. ^1^*H*-NMR (300 MHz, CDCl_3_) δ 7.99 (d, *J* = 8.7 Hz, 1H), 6.79 (dd, *J* = 8.8, 2.6 Hz, 1H), 6.65 (d, *J* = 2.5 Hz, 1H), 3.81 (s, 3H), 2.92 (t, *J* = 6.4 Hz, 2H), 2.00–1.85 (m, 2H), 1.19 (s, 6H). ^13^C-NMR (75 MHz, CDCl_3_) δ 200.95, 162.86, 145.36, 129.76, 124.49, 112.84, 111.83, 54.84, 40.78, 36.30, 25.65, 24.06.

7-Methoxy-2,2-dimethyl-3,4-dihydronaphthalen-1(2*H*)-one **8c** (yield 93%) as a yellow oil. ^1^H-NMR (300 MHz, CDCl_3_) δ 7.52 (d, *J* = 2.8 Hz, 1H), 7.13 (d, *J* = 8.4 Hz, 1H), 7.04 (dd, *J* = 8.4, 2.8 Hz, 1H), 3.83 (s, 3H), 2.91 (t, *J* = 6.3 Hz, 2H), 1.96 (t, *J* = 6.3 Hz, 2H), 1.21 (s, 6H). ^13^C-NMR (75 MHz, CDCl_3_) δ 202.71, 158.28, 135.82, 132.10, 129.78, 121.33, 109.77, 55.35, 41.36, 36.76, 30.80, 24.82, 24.28.

7-Br-2,2-dimethyl-3,4-dihydronaphthalen-1(2*H*)-one **8d** (yield 86%) as a red oil. ^1^H-NMR (300 MHz, CDCl_3_) δ 7.99 (d, *J* = 2.2 Hz, 1H), 7.37 (dd, *J* = 8.2, 2.2 Hz, 1H), 6.96 (d, *J* = 8.2 Hz, 1H), 2.78 (t, *J* = 6.3 Hz, 2H), 1.82 (t, *J* = 6.4 Hz, 2H),, 1.07 (s, 6H). ^13^C-NMR (75 MHz, CDCl_3_) δ 200.09, 140.92, 134.57, 131.85, 129.54, 129.49, 119.49, 40.32, 35.18, 24.12, 23.11.

6-Methoxy-2,2-dimethyl-2,3-dihydronaphthalene-1,4-dione (**9b**) [27,28]: To a stirred solution of 6-methoxy-2,2-dimethyl-3,4-dihydronaphthalen-1(2*H*)-one **8b** (10.0 g, 49 mmol), peroxy-*t*-butanol (*t*-BuOOH, 62.8 g, 490 mmol, 70% aqueous) and Co(acac)_2_ (1.26 g, 4.9 mmol) in 150 mL acetone at r.t. for 24 h. The mixture was then extracted with ethyl acetate (3 × 50 mL), washed with water (2 × 50 mL) and dried over anhydrous Na_2_SO_4_. Evaporation of the solvent yielded a brown oil. The residue was subjected to silica gel chromatography using PE and EtOAc (5:1) as eluent to afford 6-methoxy-2,2-dimethyl-2,3-dihydronaphthalene-1,4-dione **9b** (7.7 g, 72%) as a colorless solid. ^1^H-NMR (300 MHz, CDCl_3_) δ 8.04 (d, *J* = 8.7 Hz, 1H), 7.42 (d, *J* = 2.7 Hz, 1H), 7.23 (dd, *J* = 8.7, 2.7 Hz, 1H), 3.94 (s, 3H), 2.93 (s, 2H), 1.31 (s, 6H). ^13^C-NMR (75 MHz, CDCl_3_) δ 200.14, 196.37, 164.01, 136.94, 129.89, 127.07, 121.61, 108.31, 55.82, 52.21, 45.21, 25.90.

7-Methoxy-2,2-dimethyl-2,3-dihydronaphthalene-1,4-dione **9c** (74%) as a colorless solid. ^1^H-NMR (300 MHz, CDCl_3_) δ 7.99 (d, *J* = 8.6 Hz, 1H), 7.48 (d, *J* = 2.7 Hz, 1H), 7.21 (dd, *J* = 8.6, 2.7 Hz, 1H), 3.94 (s, 3H), 2.89 (s, 2H), 1.31 (s, 6H). ^13^C-NMR (75 MHz, CDCl_3_) δ 201.43, 194.85, 164.42, 135.81, 128.53, 128.41, 121.05, 109.85, 55.81, 51.66, 45.60, 30.84, 25.77.

7-Br-2,2-dimethyl-2,3-dihydronaphthalene-1,4-dione **9d** (69%) as a red solid. ^1^H-NMR (300 MHz, CDCl_3_) δ 8.20 (dd, *J* = 1.9, 0.6 Hz, 1H), 8.00–7.69 (m, 2H), 2.92 (s, 2H), 1.31 (s, 6H). ^13^C-NMR (75 MHz, CDCl_3_) δ 199.96, 195.16, 137.01, 134.84, 133.50, 130.59, 129.89, 127.96, 51.77, 45.77, 25.68.

#### 3.2.2. Synthesis of 13a (Pathway b in Scheme 1)

3-Amino-5,5-dimethylcyclohex-2-enone (**11**) [29]: 5,5-dimethylcyclohexane-1,3-dione **10** (7.0 g, 50 mmol) was added to a mixture of ammonium acetate (3.85 g, 50 mmol) in dry toluene (100 mL). The mixture was heated for 5 h under reflux using a Dean–Stark water separator. The red oily layer formed was then separated and recrystallized with ethyl acetate to afford 3-amino-5,5-dimethylcyclohex-2-enone **11** (5.9 g, yield 85%) as a yellow solid. ^1^H-NMR (300 MHz, DMSO-*d*_6_) δ 6.67 (s, 2H), 4.90 (s, 1H), 2.11 (s, 2H), 1.90 (s, 2H), 0.96 (s, 6H).

7,7-Dimethyl-7,8-dihydroquinolin-5(6*H*)-one (**12a**) [29]: A solution of 1,1,3,3-tetraethoxylpropane (10 mL, 40 mmol), 3-amino-5,5-dimethylcyclohex-2-enone **11** (5.6 g, 40 mmol) and a catalytical amount of *p*-toluenesulfonic acid hydrate in DMF (50 mL) was heated under reflux for 18 h (monitored by TLC). The solvent was distilled in vacuo, the residue neutralized with NaHCO_3_, extracted with EtOAc (3 × 100 mL) and dried (anhydrous Na_2_SO_4_). Column chromatography using PE and EtOAc (6:1) as eluent to afford 7,7-dimethyl-7,8-dihydroquinolin-5(6*H*)-one **12a** (2.1 g, yield 30%) as a yellow solid. ^1^H-NMR (300 MHz, CDCl_3_) δ 8.71 (d, *J* = 4.7 Hz, 1H), 8.25 (d, *J* = 7.8 Hz, 1H), 7.30 (dd, *J* = 7.8, 4.8 Hz, 1H), 3.06 (s, 2H), 2.56 (s, 2H), 1.12 (s, 6H). ^13^C-NMR (75 MHz, CDCl_3_) δ 197.41, 161.82, 153.36, 134.25, 126.90, 121.86, 51.67, 45.94, 32.60, 27.94.

7,7-Dimethyl-6,7-dihydroquinoline-5,8-dione (**13a**): The synthesis of compound **13a** was followed the same method as the synthesis of **9b** using intermediate **12a** as substrate.

7,7-Dimethyl-6,7-dihydroquinoline-5,8-dione **13a** (yield 42%) as a yellow solid. ^1^H-NMR (300 MHz, CDCl_3_) δ 9.08 (dd, *J* = 4.6, 1.7 Hz, 1H), 8.37 (dd, *J* = 7.9, 1.8 Hz, 1H), 7.67 (dd, *J* = 7.9, 4.6 Hz, 1H), 3.02 (s, 2H), 1.37 (s, 6H). ^13^C-NMR (75 MHz, CDCl_3_) δ 199.22, 195.03, 155.53, 149.62, 134.68, 131.69, 127.66, 51.84, 45.49, 25.53.

#### 3.2.3. Synthesis of **13b**–**13d** (Pathway c in Scheme 1, Use **13b** as Example)

4-(Dimethylamino)but-3-en-2-one (**15b**) [31]: To a mixture of acetone **14b** (5.8 g, 100 mmol), in xylene (200 mL), was added dimethylformamide dimethylacetal (DMF-DMA, 11.9 g, 100 mmol) and the reaction refluxed for 15 h (monitored by TLC). The xylene was distilled off and the resulting solid residue was purified by column chromatography using PE and EtOAc (6:1) as eluent to afford 4-(dimethylamino)but-3-en-2-one **15b** (4.3 g, 38%) as a yellow oil. ^1^H-NMR (300 MHz, Chloroform-*d*) δ 7.48 (d, *J* = 12.8 Hz, 1H), 5.05 (d, *J* = 12.8 Hz, 1H), 2.95 (d, *J* = 55.7 Hz, 6H), 2.09 (s, 3H).

1-(Dimethylamino)hex-1-en-3-one **15c** (25%) as a yellow oil. ^1^H-NMR (300 MHz, CDCl_3_) δ 7.19 (d, *J* = 12.7 Hz, 1H), 4.72 (d, *J* = 12.7 Hz, 1H), 2.62 (d, *J* = 27.6 Hz, 6H), 2.08–1.92 (m, 2H), 1.31 (h, *J* = 7.4 Hz, 2H), 0.61 (t, *J* = 7.4 Hz, 3H).

3-(Dimethylamino)-1-phenylprop-2-en-1-one **15d** (81%) as a yellow solid. ^1^H-NMR (300 MHz, CDCl_3_) δ 7.90 (dd, *J* = 7.9, 1.7 Hz, 2H), 7.80 (d, *J* = 12.4 Hz, 1H), 7.54–7.35 (m, 3H), 5.72 (d, *J* = 12.4 Hz, 1H), 3.13 (s, 3H), 2.93 (s, 3H).

2,7,7-Trimethyl-7,8-dihydroquinolin-5(6*H*)-one (**12b**) [31]: To a mixture of 4-(dimethylamino)but-3-en-2-one **15b** (2.5 g, 22 mmol), 5,5-dimethylcyclohexane-1,3-dione (3.1 g, 22 mmol),ammonium acetate (NH_4_Oac, 3.4 g, 44 mmol) in 50 mL of AcOH refluxed for 15 h (monitored by TLC). The resulting mixture was cooled to r.t., the solution was concentrated on rotary evaporator and then purified by column chromatography using PE and EtOAc (4:1) as eluent to afford 2,7,7-trimethyl-7,8-dihydroquinolin-5(6*H*)-one **12b** (3.4 g, 81%) as a yellow oil. ^1^H-NMR (300 MHz, CDCl_3_) δ 8.15 (d, *J* = 8.0 Hz, 1H), 7.14 (d, *J* = 8.0 Hz, 1H), 3.00 (s, 2H), 2.60 (s, 3H), 2.52 (s, 2H), 1.11 (s, 6H). ^13^C-NMR (75 MHz, CDCl_3_) δ 197.57, 163.46, 161.66, 134.66, 124.71, 121.77, 51.84, 46.26, 32.79, 28.13, 24.80.

7,7-Dimethyl-2-propyl-7,8-dihydroquinolin-5(6*H*)-one **12c** (63%) as a yellow oil. ^1^H-NMR (300 MHz, CDCl_3_) δ 7.79 (d, *J* = 8.0 Hz, 1H), 6.78 (d, *J* = 8.0 Hz, 1H), 2.66 (s, 2H), 2.52–2.37 (m, 2H), 2.16 (s, 2H), 1.43 (h, *J* = 7.4 Hz, 2H), 0.75 (s, 6H), 0.63 (t, *J* = 7.4 Hz, 3H). ^13^C-NMR (75 MHz, CDCl_3_) δ 196.59, 166.65, 161.07, 133.91, 124.30, 120.52, 51.29, 45.89, 40.12, 32.19, 27.63, 22.17, 13.25.

7,7-Dimethyl-2-phenyl-7,8-dihydroquinolin-5(6*H*)-one **12d** (92%) as a yellow solid. ^1^H-NMR (300 MHz, CDCl_3_) δ 8.31 (d, *J* = 8.2 Hz, 1H), 8.06 (dd, *J* = 7.8, 1.7 Hz, 2H), 7.71 (d, *J* = 8.2 Hz, 1H), 7.51–7.47 (m, 3H), 3.11 (s, 2H), 2.57 (s, 2H), 1.14 (s, 6H). 

2,7,7-Trimethyl-6,7-dihydroquinoline-5,8-dione (**13b**): The synthesis of compound **13b**–**13d** was followed the same method as the synthesis of **9b** using intermediate **12b**–**12d** as substrate.

2,7,7-Trimethyl-6,7-dihydroquinoline-5,8-dione **13b** (yield 57%) as a yellow solid. ^1^H-NMR (300 MHz, CDCl_3_) δ 8.24 (d, *J* = 8.1 Hz, 1H), 7.53 (d, *J* = 8.1 Hz, 1H), 2.99 (s, 2H), 2.77 (s, 3H), 1.36 (s, 6H). ^13^C-NMR (75 MHz, CDCl_3_) δ 199.54, 194.92, 165.77, 149.03, 134.68, 129.39, 127.65, 51.64, 45.37, 25.45, 25.21.

7,7-Dimethyl-2-propyl-6,7-dihydroquinoline-5,8-dione **13c** (yield 61%) as a yellow oil. ^1^H-NMR (300 MHz, CDCl_3_) δ 8.04 (d, *J* = 8.1 Hz, 1H), 7.36 (d, *J* = 8.1 Hz, 1H), 2.90–2.67 (m, 4H), 1.62 (h, *J* = 7.4 Hz, 2H), 1.15 (s, 6H), 0.79 (t, *J* = 7.3 Hz, 3H). ^13^C-NMR (75 MHz, CDCl_3_) δ 199.01, 194.49, 169.05, 148.73, 134.24, 129.20, 126.56, 51.24, 44.93, 40.34, 25.06, 22.37, 13.34.

7,7-Dimethyl-2-phenyl-6,7-dihydroquinoline-5,8-dione **13d** (yield 51%) as a yellow solid. ^1^H-NMR (300 MHz, CDCl_3_) δ 8.40 (d, *J* = 8.3 Hz, 1H), 8.22–8.15 (m, 2H), 8.08 (d, *J* = 8.3 Hz, 1H), 7.52 (dd, *J* = 5.1, 1.9 Hz, 3H), 3.02 (s, 2H), 1.39 (s, 6H). ^13^C-NMR (75 MHz, CDCl_3_) δ 199.27, 194.78, 162.64, 149.54, 137.25, 135.39, 130.57, 129.81, 129.76, 128.82, 128.41, 127.62, 123.89, 51.71, 45.43, 25.40.

#### 3.2.4. Synthesis of **17** (Pathway d in Scheme 1)

6,6-Dimethyl-6,7-dihydrobenzofuran-4(5*H*)-one (**16**) [32,33]: To a stirred ice-cooled suspension of 5,5-dimethylcyclohexane-1,3-dione **10** (14 g, 100 mmol) in water (100 mL) was added dropwise a solution of KOH (7 g, 125 mmol) in water (100 mL). Then, KI (0.3 g, 20 mmol) was added to the resulting clear solution followed by the dropwise addition of 40% aqueous chloroacetaldehyde (ClCH_2_CHO, 20 mL) over 25 min. The reaction mixture was allowed to warm to reflux and stir overnight. The reaction was quenched by the dropwise addition of 2 M HCl until acid to pH paper. The solution was extracted by EtOAc (3 × 100 mL). The combined organic layers were dried (anhydrous Na_2_SO_4_), concentrated, and vacuum distilled to afford 6,6-dimethyl-6,7-dihydrobenzofuran-4(5*H*)-one **16** (12.5 g, 76%) as a yellow oil. ^1^H-NMR (300 MHz, CDCl_3_) δ 7.25 (d, *J* = 2.0 Hz, 1H), 6.57 (d, *J* = 2.0 Hz, 1H), 2.66 (s, 2H), 2.29 (s, 2H), 1.05 (s, 6H). ^13^C-NMR (75 MHz, CDCl_3_) δ 193.53, 166.03, 142.70, 119.66, 106.07, 51.86, 37.13, 35.03, 28.31.

6,6-Dimethyl-5,6-dihydrobenzofuran-4,7-dione (**17**): The synthesis of compound **17** was followed the same method as the synthesis of **9b** using intermediate **16** as substrate.

6,6-Dimethyl-5,6-dihydrobenzofuran-4,7-dione **17** (yield 52%) as a white solid. ^1^H-NMR (300 MHz, CDCl_3_) δ 7.72 (d, *J* = 1.9 Hz, 1H), 6.80 (d, *J* = 1.9 Hz, 1H), 2.90 (s, 2H), 1.35 (s, 6H). ^13^C-NMR (75 MHz, CDCl_3_) δ 192.11, 190.88, 153.06, 148.64, 132.08, 107.55, 53.99, 46.59, 26.23.

#### 3.2.5. General Procedure for the Preparation of Intermediate **18b**–**18d**, **19a**–**19d** and **20** (Use **18b** as Example) [34]

To a stirred solution of (*Z*)-3-methylpent-2-en-4-yn-1-ol (0.88 g, 9.2 mmol) in dry THF (20 mL) was cooled to −78 °C under an atmosphere of argon. *n*-Butyl lithium (7.7 mL, 18.4 mmol, 2.4 M in hexane) was then added slowly, via syringe. The mixture was allowed to stir at −78 °C for 1 h, after which, 6-methoxy-2,2-dimethyl-2,3-dihydronaphthalene-1,4-dione **9b** (2 g, 9.2 mmol), dissolved in dry THF (10 mL) was added. The mixture was stirred for a further 1 h at −78 °C, and then the cold bath was removed. The reaction mixture was stirred at r.t. for a further 16 h. The reaction was quenched by addition of a saturated aqueous solution of NH_4_Cl. The mixture was stirred for 10 min and extracted with ethyl acetate (3 × 50 mL), washed with water (2 × 30 mL) and dried over anhydrous Na_2_SO_4_. Evaporation of the solvent yielded the desired alcohol as a yellowish oil. The residue was subjected to silica gel chromatography using PE and EtOAc (4:1) as eluent to afford (*Z*)-(1′-hydroxy-3′,3′-dimethyl-4′-oxo-7′-methoxy-tetrahydronaphthalene-one-yl)-3-methyl-pentyl-2-em-4-yn-1-ol **18b** (2.37 g, 82%) as a yellow oil. ^1^H-NMR (300 MHz, CDCl_3_) δ 7.80 (d, *J* = 8.6 Hz, 1H), 7.46 (d, *J* = 2.8 Hz, 1H), 7.13 (dd, *J* = 8.6, 2.9 Hz, 1H), 5.88 (t, *J* = 7.5 Hz, 1H), 4.27 (d, *J* = 6.7 Hz, 2H), 3.83 (s, 3H), 2.92 (d, *J* = 16.9 Hz, 1H), 2.73 (s, 1H), 2.58 (d, *J* = 17.4 Hz, 1H), 1.89 (s, 3H), 1.21 (s, 3H), 1.13 (s, 3H). ^13^C-NMR (75 MHz, CDCl_3_) δ 197.49, 159.63, 136.82, 136.36, 131.11, 129.04, 121.66, 120.16, 109.42, 94.54, 85.58, 74.29, 61.03, 55.54, 48.38, 41.60, 25.02, 23.31, 23.02.

(*Z*)-(1′-Hydroxy-3′,3′-dimethyl-4′-oxo-6′-methoxy-tetrahydronaphthalene-one-yl)-3-methyl-pentyl-2-em-4-yn-1-ol **18c** (75%) as a yellow oil. ^1^H-NMR (300 MHz, CDCl_3_) δ 7.96 (d, *J* = 8.7 Hz, 1H), 7.39 (d, *J* = 2.4 Hz, 1H), 6.90 (d, *J* = 8.7 Hz, 1H), 5.89 (t, *J* = 6.4 Hz, 1H), 4.27 (d, *J* = 6.4 Hz, 2H), 3.87 (s, 3H), 3.01–2.41 (m, 3H), 1.88 (s, 3H), 1.16 (s, 6H). ^13^C-NMR (75 MHz, CDCl_3_) δ 196.35, 164.42, 147.09, 136.57, 129.46, 123.57, 120.04, 114.19, 111.84, 94.29, 85.76, 74.60, 60.98, 55.52, 48.48, 41.51, 30.86, 24.94, 22.97.

(*Z*)-(1′-Hydroxy-3′,3′-dimethyl-4′-oxo-6′-Br-tetrahydronaphthalene-one-yl)-3-methyl-pentyl-2-em-4-yn-1-ol **18d** (70%) as a yellow oil. ^1^H-NMR (300 MHz, CDCl_3_) δ 8.06 (d, *J* = 1.9 Hz, 1H), 7.85 (d, *J* = 8.3 Hz, 1H), 7.56 (dd, *J* = 8.3, 1.9 Hz, 1H), 5.92 (t, *J* = 6.8 Hz, 1H), 4.27 (d, *J* = 6.8 Hz, 2H), 4.03 (s, 1H), 2.85 (d, *J* = 18.7 Hz, 1H), 2.64 (d, *J* = 23.1 Hz, 2H), 1.89 (s, 3H), 1.17 (s, 6H). ^13^C-NMR (75 MHz, CDCl_3_) δ 196.68, 146.17, 136.66, 131.84, 130.46, 129.55, 128.80, 128.58, 120.18, 93.72, 86.38, 74.11, 60.94, 48.57, 41.62, 24.92, 22.95.

(*Z*)-(5′-Hydroxy-7′,7′-dimethyl-8′-oxo-tetrahydroquinolin-one-yl)-3-methyl-pentyl-2-em-4-yn-1-ol **19a** (yield 79%) as a yellow oil. ^1^H-NMR (300 MHz, CDCl_3_) δ 8.77 (dd, *J* = 4.8, 1.8 Hz, 1H), 8.30 (dd, *J* = 7.8, 1.8 Hz, 1H), 7.45 (dd, *J* = 7.8, 4.8 Hz, 1H), 5.85 (t, *J* = 6.6 Hz, 1H), 5.48 (s, 1H), 4.14 (d, *J* = 6.5 Hz, 2H), 3.00 (d, *J* = 17.4 Hz, 2H), 2.68 (d, *J* = 17.4 Hz, 1H), 1.79 (s, 3H), 1.39 (s, 3H), 1.02 (s, 3H). ^13^C-NMR (75 MHz, CDCl_3_) δ 196.02, 161.46, 153.11, 136.96, 135.23, 124.98, 123.67, 119.37, 93.94, 85.72, 73.93, 60.68, 50.01, 40.70, 24.67, 22.63, 21.14.

(*Z*)-(5′-Hydroxy-2′,7′,7′-trimethyl-8′-oxo-tetrahydroquinolin-one-yl)-3-methyl-pentyl-2-em-4-yn-1-ol **19b** (yield 85%) as a yellow oil. ^1^H-NMR (300 MHz, CDCl_3_) δ 8.17 (d, *J* = 7.9 Hz, 1H), 7.28 (d, *J* = 8.0 Hz, 1H), 5.84 (t, *J* = 6.6 Hz, 1H), 5.35 (s, 1H), 4.16 (d, *J* = 6.6 Hz, 2H), 3.00 (d, *J* = 17.5 Hz, 1H), 2.66 (s, 3H), 2.59 (d, *J* = 17.5 Hz, 2H), 1.80 (s, 3H), 1.40 (s, 3H), 0.97 (s, 3H). ^13^C-NMR (75 MHz, CDCl_3_) δ 195.87, 163.50, 160.80, 136.79, 135.44, 123.43, 122.57, 119.43, 94.30, 85.23, 73.63, 60.86, 50.24, 40.79, 24.67, 24.66, 22.68, 20.93.

(*Z*)-(5′-Hydroxy-7′,7′-dimethyl-2′-propyl-8′-oxo-tetrahydroquinolin-one-yl)-3-methyl-pentyl-2-em-4-yn-1-ol **19c** (yield 73%) as a yellow oil. ^1^H-NMR (300 MHz, CDCl_3_) δ 8.10 (d, *J* = 7.9 Hz, 1H), 7.19 (d, *J* = 7.9 Hz, 1H), 5.75 (t, *J* = 6.1 Hz, 1H), 5.31 (s, 1H), 4.09 (d, *J* = 6.3 Hz, 2H), 2.93 (d, *J* = 17.4 Hz, 1H), 2.80 (d, *J* = 7.3 Hz, 2H), 2.50 (d, *J* = 17.5 Hz, 1H), 1.81–1.65 (m, 5H), 1.32 (s, 3H), 0.92–0.86 (m, 6H). ^13^C-NMR (75 MHz, CDCl_3_) δ 195.60, 166.92, 160.52, 136.72, 135.12, 122.56, 122.38, 118.79, 94.04, 84.93, 73.34, 60.55, 50.03, 40.45, 39.82, 24.42, 22.39, 21.98, 20.59, 13.30.

(*Z*)-(5′-Hydroxy-7′,7′-dimethyl-2′-phenyl-8′-oxo-tetrahydroquinolin-one-yl)-3-methyl-pentyl-2-em-4-yn-1-ol **19d** (yield 79%) as a yellow oil. ^1^H-NMR (300 MHz, CDCl_3_) δ 8.35 (d, *J* = 8.2 Hz, 1H), 8.20–8.02 (m, 2H), 7.87 (d, *J* = 8.2 Hz, 1H), 7.53 (qd, *J* = 4.6, 1.6 Hz, 3H), 5.82 (t, *J* = 7.4 Hz, 1H), 5.34 (s, 1H), 4.17 (s, 2H), 3.08 (d, *J* = 17.7 Hz, 1H), 2.62 (d, *J* = 17.6 Hz, 1H), 1.80 (s, 3H), 1.45 (s, 3H), 1.00 (s, 3H). ^13^C-NMR (75 MHz, CDCl_3_) δ 195.74, 161.32, 160.49, 137.29, 136.59, 136.36, 130.62, 128.99, 127.50, 123.46, 120.24, 119.88, 94.60, 85.30, 73.89, 61.28, 50.61, 40.97, 24.81, 22.87, 21.00.

(*Z*)-(4′-Hydroxy-6′,6′-dimethyl-7′-oxo-tetrahydrobenzofuran-one-yl)-3-methyl-pentyl-2-em-4-yn-1-ol **20** (yield 90%) as a yellow oil. ^1^H-NMR (300 MHz, CDCl_3_) δ 7.42 (d, *J* = 2.0 Hz, 1H), 6.68 (d, *J* = 2.0 Hz, 1H), 5.95 (t, *J* = 6.8 Hz, 1H), 4.31 (d, *J* = 6.7 Hz, 2H), 2.80 (d, *J* = 16.4 Hz, 1H), 2.45 (d, *J* = 16.5 Hz, 1H), 1.88 (s, 3H), 1.21 (d, *J* = 12.0 Hz, 6H). ^13^C-NMR (75 MHz, CDCl_3_) δ 193.65, 163.80, 143.91, 137.11, 119.66, 119.21, 106.44, 90.38, 85.67, 70.16, 60.77, 49.40, 43.91, 24.79, 22.67, 22.55.

#### 3.2.6. General Procedure for the Preparation of Intermediate **21b**−**21d**, **22a**−**22d** and **23** (Use **21b** as Example)

To a stirred solution of (*Z*)-(1′-hydroxy-3′,3′-dimethyl-4′-oxo-7′-methoxy-tetrahydronaphthalene-one-yl)-3-methyl-pentyl-2-em-4-yn-1-ol **18b** (1 g, 3.2 mmol) in dry THF (20 mL) was cooled to 0 °C and Red-Al reagent (2.9 mL, 9.6 mmol, 3.3 M in toluene) added dropwise via syringe. After 4 h stirring at r.t., the reaction was quenched by slow addition of saturate brine (10 mL) and extracted with diethyl ether (3 × 30 mL). The organic phase was washed with water (2 × 20 mL) and dried over anhydrous Na_2_SO_4_ and filtered, filtrate concentrated under reduced pressure. The residue was subjected to silica gel chromatography using CH_2_Cl_2_ and methanol (25:1) as eluent to afford (1*E*,3*Z*)-(1′,4′-dihydroxy-3′,3′-dimethyl-7′-methoxy-tetraloneyl)-3-methyl-pentyl-2-ene-4-yn-1-ol **21b** (0.57 g, 56%) as a white solid. ^1^H-NMR (300 MHz, MeOD) δ 7.32 (d, *J* = 8.7 Hz, 1H), 7.06 (d, *J* = 2.5 Hz, 1H), 6.81 (dd, *J* = 8.7, 2.7 Hz, 1H), 6.31 (d, *J* = 15.6 Hz, 1H), 6.05 (d, *J* = 15.7 Hz, 1H), 5.44 (t, *J* = 6.9 Hz, 1H), 4.76 (dd, *J* = 9.9, 6.8 Hz, 1H), 4.09 (d, *J* = 7.1 Hz, 2H), 3.79 (s, 3H), 1.91 (dd, *J* = 13.3, 6.7 Hz, 1H), 1.82 (d, *J* = 13.3 Hz, 4H), 0.99 (d, *J* = 5.8 Hz, 6H). ^13^C-NMR (75 MHz, MeOD) δ 160.23, 141.14, 138.07, 134.97, 134.39, 130.26, 129.55, 126.56, 115.18, 111.87, 79.59, 67.32, 58.48, 55.63, 44.95, 39.99, 25.70, 23.24, 20.82.

(1*E*,3*Z*)-(1′,4′-Dihydroxy-3′,3′-dimethyl-6′-methoxy-tetraloneyl)-3-methyl-pentyl-2-ene-4-yn-1-ol **21c** (49%) as a white solid. ^1^H-NMR (300 MHz, MeOD) δ 7.42 (d, *J* = 8.1 Hz, 1H), 6.98 (d, *J* = 2.7 Hz, 1H), 6.83 (dd, *J* = 8.6, 2.7 Hz, 1H), 6.37 (d, *J* = 15.7 Hz, 1H), 6.06 (d, *J* = 15.5 Hz, 1H), 5.46 (t, *J* = 6.8 Hz, 1H), 4.75 (dd, *J* = 9.7, 7.0 Hz, 1H), 4.10 (dd, *J* = 6.5, 2.8 Hz, 2H), 3.75 (s, 3H), 1.91 (dd, *J* = 13.4, 6.9 Hz, 1H), 1.87–1.72 (m, 4H), 0.99 (d, *J* = 10.5 Hz, 6H). ^13^C-NMR (75 MHz, MeOD) δ 160.48, 143.82, 137.72, 134.99, 132.00, 129.69, 129.51, 126.76, 114.75, 112.80, 79.89, 67.00, 58.47, 55.61, 45.06, 40.26, 25.65, 23.10, 20.79.

(1*E*,3*Z*)-(1′,4′-Dihydroxy-3′,3′-dimethyl-6′-Br-tetraloneyl)-3-methyl-pentyl-2-ene-4-yn-1-ol **21d** (45%) as a yellow oil. ^1^H-NMR (300 MHz, CDCl_3_) δ 7.59–7.50 (m, 1H), 7.50–7.39 (m, 1H), 7.36–7.26 (m, 2H), 6.29 (d, *J* = 15.7 Hz, 1H), 6.03 (d, *J* = 15.7 Hz, 1H), 5.51 (t, *J* = 7.1 Hz, 1H), 4.95–4.77 (m, 1H), 4.06 (d, *J* = 7.8 Hz, 2H), 2.17 (s, 2H), 2.03 (q, *J* = 6.9 Hz, 2H), 1.81 (s, 3H), 1.01 (d, *J* = 15.2 Hz, 6H). ^13^C-NMR (75 MHz, CDCl_3_) δ 140.59, 138.16, 135.73, 135.22, 128.17, 128.03, 127.62, 127.31, 127.24, 126.71, 78.87, 66.64, 57.97, 43.85, 39.11, 25.05, 22.51, 20.48.

(1*E*,3*Z*)-(5′,8′-Dihydroxy-7′,7′-dimethyl-tetrahyroquinolinyl)-3-methyl-pentyl-2-ene-4-yn-1-ol **22a** (yield 12%) as a white solid. ^1^H-NMR (300 MHz, CDCl_3_) δ 8.54–8.45 (m, 1H), 7.95 (dd, *J* = 7.8, 1.4 Hz, 1H), 7.33–7.23 (m, 1H), 6.06 (m, 2H), 5.51 (t, *J* = 7.7 Hz, 1H), 4.99–4.67 (m, 1H), 4.02 (dd, *J* = 7.2, 3.9 Hz, 2H), 3.00 (d, *J* = 16.9 Hz, 1H), 2.65 (d, *J* = 17.5 Hz, 1H), 1.80 (s, 3H), 1.10 (s, 3H), 0.97 (s, 3H). ^13^C-NMR (75 MHz, CDCl_3_) δ 157.52, 152.85, 148.02, 135.90, 135.14, 132.74, 127.71, 126.82, 122.41, 78.03, 65.54, 57.50, 43.22, 38.94, 24.60, 21.88, 20.01.

(1*E*,3*Z*)-(5′,8′-Dihydroxy-2′,7′,7′-trimethyl-tetrahyroquinolin-yl)-3-methyl-pentyl-2-ene-4-yn-1-ol **22b** (yield 43%) as a yellow oil. ^1^H-NMR (300 MHz, CDCl_3_) δ 7.77 (d, *J* = 7.9 Hz, 1H), 7.11 (d, *J* = 7.9 Hz, 1H), 6.45 (d, *J* = 15.6 Hz, 1H), 5.87 (d, *J* = 15.6 Hz, 1H), 5.51 (t, *J* = 7.0 Hz, 1H), 4.86 (dd, *J* = 6.9, 3.2 Hz, 1H), 4.18 (dd, *J* = 6.6, 3.2 Hz, 2H), 2.51 (s, 3H), 2.20 (dd, *J* = 14.9, 6.9 Hz, 1H), 1.88–1.82 (m, 1H), 1.79 (s, 3H), 1.02 (d, *J* = 8.3 Hz, 6H). ^13^C-NMR (75 MHz, CDCl_3_) δ 157.03, 156.50, 136.95, 134.22, 134.16, 128.87, 128.01, 125.70, 122.30, 77.53, 64.78, 57.87, 50.20, 42.65, 36.27, 24.22, 23.74, 20.32.

(1*E*,3*Z*)-(5′,8′-Dihydroxy-7′,7′-dimethyl-2′-propyl-tetrahyroquinolin-yl)-3-methyl-pentyl-2-ene-4-yn-1-ol **22c** (yield 26%) as a yellow oil. ^1^H-NMR (300 MHz, CDCl_3_) δ 7.82 (d, *J* = 7.9 Hz, 1H), 7.10 (d, *J* = 7.9 Hz, 1H), 6.15–5.91 (m, 2H), 5.51 (t, *J* = 7.3 Hz, 1H), 4.89 (s, 1H), 4.86–4.75 (m, 1H), 4.01 (d, *J* = 8.8 Hz, 2H), 2.77–2.72 (m, 2H), 2.03–1.84 (m, 2H), 1.81 (s, 3H), 1.77–1.69 (m, 2H), 1.11 (s, 3H), 0.94 (d, *J* = 7.3 Hz, 6H). ^13^C-NMR (75 MHz, CDCl_3_) δ 160.78, 156.37, 136.29, 135.98, 135.36, 129.53, 127.44, 126.81, 121.49, 77.78, 65.59, 57.52, 43.41, 39.39, 39.04, 24.60, 22.35, 21.85, 20.09, 13.38.

(1*E*,3*Z*)-(5′,8′-Dihydroxy-7′,7′-dimethyl-2′-phenyl-tetrahyroquinolin-yl)-3-methyl-pentyl-2-ene-4-yn-1-ol **22d** (yield 65%) as a yellow oil. ^1^H-NMR (300 MHz, CDCl_3_) δ 7.98 (d, *J* = 7.9 Hz, 3H), 7.69 (d, *J* = 8.1 Hz, 1H), 7.43 (m, 3H), 6.08 (s, 2H), 5.48 (t, *J* = 7.1 Hz, 1H), 4.93 (s, 1H), 4.90–4.77 (m, 1H), 3.99 (dd, *J* = 6.9, 3.6 Hz, 2H), 2.29 (t, *J* = 6.0 Hz, 1H), 1.97 (m, 3H), 1.79 (s, 3H), 1.13 (s, 3H), 0.99 (s, 3H). ^13^C-NMR (75 MHz, CDCl_3_) δ 160.43, 157.42, 155.70, 138.38, 137.04, 136.46, 135.70, 131.33, 129.21, 128.68, 127.80, 127.31, 126.80, 125.06, 119.37, 78.31, 65.87, 57.89, 43.64, 39.48, 24.88, 22.22, 20.34.

(1*E*,3*Z*)-(4′,7′-Dihydroxy-6′,6′-dimethyl-tetrahyrobenzofuran-yl)-3-methyl-pentyl-2-ene-4-yn-1-ol **23** (yield 39%) as a yellow oil. ^1^H-NMR (300 MHz, CDCl_3_) δ 7.35 (d, *J* = 1.9 Hz, 1H), 6.47 (d, *J* = 1.9 Hz, 1H), 6.30 (d, *J* = 15.8 Hz, 1H), 5.91 (d, *J* = 15.7 Hz, 1H), 5.57 (t, *J* = 7.2 Hz, 1H), 4.74 (dd, *J* = 8.3, 5.8 Hz, 1H), 4.12 (qd, *J* = 12.6, 6.9 Hz, 2H), 2.53 (s, 1H), 1.95 (dd, *J* = 13.5, 5.8 Hz, 1H), 1.85 (s, 3H), 1.76–1.55 (m, 2H), 1.03 (d, *J* = 5.1 Hz, 6H). ^13^C-NMR (75 MHz, CDCl_3_) δ 151.59, 143.03, 134.79, 132.52, 128.70, 127.27, 121.86, 108.80, 76.08, 63.34, 57.96, 45.40, 41.37, 24.29, 22.29, 20.46.

#### 3.2.7. General Procedure for the Preparation of Title Compounds **4b**–**4d**, **5a**–**5d**, **6**, **24a**–**24d**, **25a**, **25b**, **25d** and **26** (Use **4b** as Example)

To a stirred solution of (1*E*,3*Z*)-(1′,4′-dihydroxy-3′,3′-dimethyl-7′-methoxy-tetraloneyl)-3-methyl-pentyl-2-ene-4-yn-1-ol **21b** (0.5 g, 1.57 mmol), Dess-Martin periodinane (DMP, 1.32 g, 3.14 mmol) in 30 mL CH_2_Cl_2_ at r.t. for 2 h. After added 5 mL aqueous Na_2_S_2_O_3_ solution and 10 mL aqueous NaHCO_3_ solution, the resulting mixture, which was stirred for 20 min, was extracted repeatedly with CH_2_Cl_2_ (3 × 30 mL). The collected organic extracts were washed with saturate aqueous brine solution, dried, and then concentrated. The crude aldehyde was dissolved in 20 mL solvent (*t*-BuOH:H_2_O = 3:1 in volume), stirred with 2-methyl-2-butene (2.2 g, 31.4 mmol, 90%), NaClO_2_ (1.67 g, 15.7 mmol, 85%) and NaH_2_PO_4_·2H_2_O (0.97 g, 6.28 mmol) at r.t. for 30 min. Extracted repeatedly with EtOAc (3 × 30 mL). The collected organic extracts were washed with saturate aqueous brine solution, dried, and concentrated under reduced pressure afford crude product. The residue was subjected to silica gel chromatography using CH_2_Cl_2_ and methanol (20:1) as eluent to afford 7′-OMe-*iso*-PhABA **4b** (0.42 g, yield 81% over two steps) as a white solid. ^1^H-NMR (300 MHz, MeOD) δ 7.65 (d, *J* = 16.4 Hz, 1H), 7.50 (d, *J* = 8.7 Hz, 1H), 7.45 (d, *J* = 2.8 Hz, 1H), 7.20 (dd, *J* = 8.7, 2.9 Hz, 1H), 6.48 (d, *J* = 16.0 Hz, 1H), 5.71 (s, 1H), 3.85 (s, 3H), 2.72 (d, *J* = 16.8 Hz, 1H), 2.59 (d, *J* = 17.5 Hz, 1H), 2.04 (d, *J* = 1.2 Hz, 3H), 1.08 (s, 3H), 1.04 (s, 3H). ^13^C-NMR (75 MHz, MeOD) δ 199.70, 169.46, 160.69, 151.21, 140.82, 140.37, 133.46, 130.84, 129.57, 122.49, 119.23, 109.90, 78.80, 55.92, 50.91, 42.38, 24.68, 23.95, 21.31. HR-MS (ESI) calcd. for C_19_H_22_O_5_ (M + NH_4_)^+^ 348.18055, measured 348.18057.

6′-OMe-*iso*-PhABA **4c** (yield 83% over two steps) as a white solid. ^1^H-NMR (300 MHz, CDCl_3_) δ 8.00 (d, *J* = 8.7 Hz, 1H), 7.75 (d, *J* = 15.8 Hz, 1H), 7.06 (d, *J* = 2.2 Hz, 1H), 6.88 (dd, *J* = 8.7, 2.5 Hz, 1H), 6.41 (d, *J* = 16.0 Hz, 1H), 5.72 (s, 1H), 3.83 (s, 3H), 2.70 (d, *J* = 16.2 Hz, 1H), 2.56 (d, *J* = 17.3 Hz, 1H), 2.02 (s, 3H), 1.08 (d, *J* = 11.6 Hz, 6H). ^13^C-NMR (75 MHz, CDCl_3_) δ 196.35, 170.87, 164.62, 151.87, 148.60, 139.22, 129.38, 128.33, 124.51, 117.74, 113.90, 111.79, 78.48, 55.51, 49.61, 41.12, 24.25, 23.38, 21.40. HR-MS (ESI) calcd. for C_19_H_22_O_5_ (M + H)^+^ 331.15400, measured 331.15402.

6′-Br-*iso*-PhABA **4d** (yield 72% over two steps) as a yellow oil. ^1^H-NMR (300 MHz, CDCl_3_) δ 8.05 (d, *J* = 9.1 Hz, 1H), 7.58 (dt, *J* = 13.5, 7.2 Hz, 2H), 7.50–7.36 (m, 1H), 6.94 (d, *J* = 15.3 Hz, 1H), 6.14 (d, *J* = 15.8 Hz, 1H), 5.98 (s, 1H), 2.83 (d, *J* = 17.8 Hz, 1H), 2.60 (d, *J* = 16.9 Hz, 1H), 2.04 (s, 1H), 1.87 (s, 3H), 1.08 (d, *J* = 4.4 Hz, 6H). ^13^C-NMR (75 MHz, CDCl_3_) δ197.21, 169.75, 145.90, 134.47, 134.44, 133.14, 130.98, 128.18, 127.13, 126.72, 126.35, 116.39, 78.21, 49.79, 40.93, 24.33, 23.40, 18.25. HR-MS (ESI) calcd. for C_18_H_19_BrO_4_ (M + H)^+^ 396.08050, measured 396.08035.

*iso*-PyABA **5a** (yield 78% over two steps) as colorless oil. ^1^H-NMR (300 MHz, CDCl_3_) δ 8.73 (dd, *J* = 4.8, 1.8 Hz, 1H), 8.39 (dd, *J* = 7.8, 1.8 Hz, 1H), 7.89 (d, *J* = 15.6 Hz, 1H), 7.48 (dd, *J* = 7.8, 4.9 Hz, 1H), 6.39 (d, *J* = 16.0 Hz, 1H), 5.70 (s, 1H), 2.87 (d, *J* = 18.7 Hz, 1H), 2.66 (d, *J* = 17.3 Hz, 1H), 2.01 (s, 3H), 1.15 (s, 3H), 1.11 (s, 3H). ^13^C-NMR (75 MHz, CDCl_3_) δ 196.24, 169.30, 163.28, 152.65, 150.54, 137.97, 135.20, 128.66, 126.25, 123.29, 117.66, 77.94, 49.44, 39.98, 23.92, 22.94, 20.68. HR-MS (ESI) calcd. for C_17_H_19_NO_4_ (M + H)^+^ 302.1387, measured 302.1387.

2′-Me-*iso*-PyABA **5b** (yield 71% over two steps) as a yellow oil. ^1^H-NMR (300 MHz, CDCl_3_) δ 8.23 (d, *J* = 8.0 Hz, 1H), 7.64 (d, *J* = 15.8 Hz, 1H), 7.28 (d, *J* = 8.0 Hz, 1H), 6.38 (d, *J* = 16.1 Hz, 1H), 5.68 (s, 1H), 2.68 (s, 2H), 2.63 (s, 3H), 1.99 (s, 3H), 1.16 (s, 3H), 1.05 (s, 3H). ^13^C-NMR (75 MHz, CDCl_3_) δ 196.04, 169.66, 163.45, 162.46, 150.81, 138.72, 135.02, 128.48, 123.65, 123.04, 117.58, 77.62, 49.54, 39.99, 24.26, 23.78, 22.85, 20.71. HR-MS (ESI) calcd. for C_18_H_21_NO_4_ (M + H)^+^ 316.1543, measured 316.1545.

2′-Pr-*iso*-PyABA **5c** (yield 87% over two steps) as a yellow oil. ^1^H-NMR (300 MHz, CDCl_3_) δ 8.24 (d, *J* = 8.0 Hz, 1H), 7.53 (d, *J* = 15.6 Hz, 1H), 7.27 (d, *J* = 6.0 Hz, 1H), 6.39 (d, *J* = 16.1 Hz, 1H), 5.68 (s, 1H), 2.90–2.80 (m, 2H), 2.77–2.56 (m, 2H), 1.99 (s, 3H), 1.83–1.72 (m, 2H), 1.20 (s, 3H), 1.04 (s, 3H), 0.95 (d, *J* = 7.4 Hz, 3H). ^13^C-NMR (75 MHz, CDCl_3_) δ 196.03, 169.79, 167.22, 162.25, 151.00, 139.08, 134.89, 128.34, 123.72, 122.34, 117.47, 77.59, 49.67, 39.97, 39.86, 23.77, 22.83, 22.15, 20.75, 13.37. HR-MS (ESI) calcd. for C_20_H_25_NO_4_ (M + H)^+^ 344.1856, measured 316.1854.

2′-Ph-*iso*-PyABA **5d** (yield 80% over two steps) as a yellow solid. ^1^H-NMR (300 MHz, CDCl_3_) δ 9.06 (s, 1H), 8.40 (d, *J* = 8.2 Hz, 1H), 8.08 (dd, *J* = 7.6, 2.0 Hz, 2H), 7.88 (d, *J* = 8.2 Hz, 1H), 7.57 (d, *J* = 15.8 Hz, 1H), 7.53–7.40 (m, 3H), 6.44 (d, *J* = 16.1 Hz, 1H), 5.67 (s, 1H), 5.13 (s, 1H), 2.82 (d, *J* = 18.2 Hz, 1H), 2.60 (d, *J* = 18.0 Hz, 1H), 1.99 (s, 3H), 1.26 (s, 3H), 1.08 (s, 3H). ^13^C-NMR (75 MHz, CDCl_3_) δ 196.03, 170.91, 162.86, 160.65, 151.65, 139.57, 137.36, 135.89, 130.39, 128.82, 128.76, 127.50, 124.52, 119.91, 117.83, 78.17, 50.06, 40.28, 24.03, 23.22, 21.09. HR-MS (ESI) calcd. for C_23_H_23_NO_4_ (M + H)^+^ 378.17000, measured 378.16980.

*iso*-FrABA **6** (yield 86% over two steps) as a yellow solid. ^1^H-NMR (300 MHz, MeOD) δ 7.68 (d, *J* = 16.4 Hz, 1H), 7.58 (s, 1H), 6.67 (s, 1H), 6.32 (d, *J* = 16.1 Hz, 1H), 5.75 (s, 1H), 3.34 (s, 1H), 2.62 (d, *J* = 17.3 Hz, 1H), 2.44 (d, *J* = 15.9 Hz, 1H), 2.04 (s, 3H), 1.07 (d, *J* = 11.6 Hz, 6H). ^13^C-NMR (75 MHz, MeOD) δ 196.03, 168.17, 150.56, 145.84, 137.65, 136.47, 130.40, 121.68, 119.99, 106.84, 76.32, 51.84, 45.09, 24.18, 23.28, 21.11. HR-MS (ESI) calcd. for C_16_H_18_O_5_ (M + Na)^+^ 313.1046, measured 313.1048.

Acetylenic *iso*-PhABA **24a** (yield 84% over two steps) as a yellow oil. ^1^H-NMR (300 MHz, CDCl_3_) δ 7.98 (d, *J* = 7.74 Hz, 2H), 7.60 (t, *J* = 7.50 Hz, 1H), 7.41 (t, J = 7.44 Hz, 1H), 6.05 (s, 1H), 2.77 (m, 2H), 2.10 (s, 3H), 1.17 (s, 6H). ^13^C-NMR (75 MHz, CDCl_3_) δ 197.5, 169.2, 143.7, 136.5, 134.3, 130.0, 128.6, 127.5, 126.8, 124.4, 101.8, 86.1, 74.7, 48.07, 41.5, 25.1, 24.8. HR-MS (ESI) calcd. for C_18_H_18_O_4_ (M + Na)^+^ 321.1097, measured 321.1096.

Acetylenic 7′-OMe-*iso*-PhABA **24b** (yield 71% over two steps) as a yellow solid. ^1^H-NMR (300 MHz, CDCl_3_) δ 7.65 (d, *J* = 8.7 Hz, 1H), 7.51 (d, *J* = 2.9 Hz, 1H), 7.18 (dd, *J* = 8.7, 2.9 Hz, 1H), 6.09 (d, *J* = 1.5 Hz, 1H), 4.67 (s, 1H), 3.88 (s, 3H), 2.90 (d, *J* = 18.1 Hz, 1H), 2.60 (d, *J* = 17.5 Hz, 1H), 2.44 (s, 3H), 1.23 (s, 3H), 1.10 (s, 3H). ^13^C-NMR (75 MHz, CDCl_3_) δ 196.65, 165.16, 159.67, 156.72, 146.97, 136.16, 133.76, 129.19, 125.58, 121.79, 118.97, 108.31, 83.52, 55.54, 49.81, 43.32, 25.19, 24.72, 18.37. HR-MS (ESI) calcd. for C_19_H_20_O_5_ (M + H)^+^ 329.13835, measured 329.13821.

Acetylenic 6′-OMe-*iso*-PhABA **24c** (yield 85% over two steps) as a yellow solid. ^1^H-NMR (300 MHz, CDCl_3_) δ 7.95 (d, *J* = 8.7 Hz, 1H), 7.47 (s, 1H), 6.87 (dd, *J* = 8.7, 2.1 Hz, 1H), 6.05 (s, 1H), 3.87 (s, 3H), 2.82 (d, *J* = 24.2 Hz, 4H), 2.07 (s, 3H), 1.17 (d, *J* = 8.6 Hz, 6H). ^13^C-NMR (75 MHz, CDCl_3_) δ 196.15, 164.44, 146.40, 129.49, 124.76, 124.70, 123.71, 114.57, 111.89, 111.83, 101.62, 86.20, 74.83, 55.61, 48.52, 41.64, 29.68, 25.12, 24.84. HR-MS (ESI) calcd. for C_19_H_20_O_5_ (M + H)^+^ 329.13835, measured 329.13828.

Acetylenic 6′-Br-*iso*-PhABA **24d** (yield 79% over two steps) as a yellow oil. ^1^H-NMR (300 MHz, CDCl_3_) δ 8.13 (s, 1H), 7.84 (d, *J* = 8.3 Hz, 1H), 7.54 (dd, *J* = 8.3, 1.9 Hz, 1H), 6.05 (d, *J* = 1.8 Hz, 1H), 2.79 (s, 2H), 2.08 (s, 3H), 1.17 (d, *J* = 17.4 Hz, 6H). ^13^C-NMR (75 MHz, CDCl_3_) δ 195.72, 168.33, 144.62, 135.17, 130.93, 129.60, 128.65, 127.87, 127.63, 123.85, 99.99, 85.67, 73.33, 47.61, 40.80, 24.13, 23.75, 21.66. HR-MS (ESI) calcd. for C_18_H_17_BrO_4_ (M + H)^+^ 394.06485, measured 394.06485.

Acetylenic *iso*-PyABA **25a** (yield 77% over two steps) as a yellow oil. ^1^H-NMR (300 MHz, CDCl_3_) δ 8.74 (dd, *J* = 4.8, 1.8 Hz, 1H), 8.32 (dd, *J* = 7.8, 1.8 Hz, 1H), 7.45 (dd, *J* = 7.8, 4.8 Hz, 1H), 5.84 (s, 1H), 5.60 (s, 1H), 5.07 (s, 1H), 2.73 (d, *J* = 17.4 Hz, 1H), 2.63 (d, *J* = 17.1 Hz, 1H), 2.15 (s, 3H), 1.28 (s, 3H), 1.05 (s, 3H). ^13^C-NMR (75 MHz, CDCl_3_) δ 195.96, 167.91, 162.11, 155.35, 153.02, 149.76, 134.68, 126.88, 123.78, 116.39, 113.15, 77.20, 49.64, 41.50, 24.31, 22.68, 11.95. HR-MS (ESI) calcd. for C_17_H_17_NO_4_ (M + H)^+^ 300.1230, measured 300.1230.

Acetylenic 2′-Me-*iso*-PyABA **25b** (yield 90% over two steps) as a yellow oil. ^1^H-NMR (300 MHz, CDCl_3_) δ 8.20 (d, *J* = 8.0 Hz, 1H), 7.27 (d, *J* = 7.7 Hz, 3H), 5.84 (s, 1H), 5.57 (s, 1H), 5.24 (s, 1H), 2.71 (d, *J* = 16.9 Hz, 1H), 2.62 (s, 3H), 2.56 (d, *J* = 17.5 Hz, 1H), 2.13 (s, 3H), 1.28 (s, 3H), 1.03 (s, 3H). ^13^C-NMR (75 MHz, CDCl_3_) δ 195.83, 168.11, 163.25, 161.47, 155.39, 149.63, 134.89, 124.46, 123.47, 116.33, 113.37, 76.91, 49.68, 41.47, 24.81, 24.31, 22.58, 11.93. HR-MS (ESI) calcd. for C_18_H_19_NO_4_ (M + H)^+^ 314.13868, measured 314.13858.

Acetylenic 2′-Ph-*iso*-PyABA **25d** (yield 75% over two steps) as a yellow oil. ^1^H-NMR (300 MHz, CDCl_3_) δ 8.35 (d, *J* = 8.2 Hz, 1H), 8.11 (dd, *J* = 7.4, 2.0 Hz, 2H), 7.85 (d, *J* = 8.2 Hz, 1H), 7.63–7.40 (m, 3H), 5.97 (s, 1H), 5.59 (s, 1H), 3.38 (d, *J* = 17.6 Hz, 1H), 2.59 (d, *J* = 17.7 Hz, 1H), 2.01 (s, 3H), 1.47 (s, 3H), 0.99 (s, 3H). ^13^C-NMR (75 MHz, CDCl_3_) δ 195.97, 168.91, 160.65, 160.24, 137.27, 136.37, 136.16, 130.41, 128.83, 127.44, 124.34, 123.67, 120.23, 101.71, 85.06, 74.04, 50.09, 41.24, 25.03, 24.50, 20.99. HR-MS (ESI) calcd. for C_23_H_21_NO_4_ (M + H)^+^ 376.15433, measured 376.15414.

Acetylenic *iso*-FrABA **26** (yield 89% over two steps) as a yellow oil. ^1^H-NMR (300 MHz, CDCl_3_) δ 7.46 (d, *J* = 1.7 Hz, 1H), 6.67 (d, *J* = 1.7 Hz, 1H), 6.10 (s, 1H), 5.78 (s, 2H), 2.75 (d, *J* = 16.9 Hz, 1H), 2.58 (d, *J* = 16.5 Hz, 1H), 2.08 (s, 3H), 1.23 (d, *J* = 6.1 Hz, 6H). ^13^C-NMR (75 MHz, CDCl_3_) δ 193.42, 163.13, 144.19, 141.58, 132.98, 127.89, 119.68, 106.58, 97.64, 86.13, 70.69, 49.85, 44.28, 24.91, 24.62, 22.42. HR-MS (ESI) calcd. for C_16_H_16_O_5_ (M + H)^+^ 289.1071, measured 289.1073.

### 3.3. Bioassays

#### 3.3.1. Arabidopsis Seed Germination

Twenty-five seeds of Arabidopsis (Columbia wild type) were sterilized successively with 70% (*v*/*v*) ethanol for 30 min and were washed with sterile water. The sterilized seeds were soaked in about 400 μL of a test solution and incubated in the dark for 3 days at 4 °C. The vernalized seeds in the test solution were transferred to plates in which two sheets of filter paper soaked in 2 mL of water had been placed and allowed to germinate under day for 16 h and night for 8 h at 22 °C. The percentage of seeds with an emerged radicle was calculated. All of the tests were conducted thrice. All of the IC_50_ values were calculated by IBM SPSS Statistics (IBM Corporation, Armonk, NY, USA).

#### 3.3.2. Lettuce Seed Germination

Fifty seeds of lettuce (*Lactuca sativa* L.cv.) were soaked in about 1 mL of a test solution in the dark for 1 day at 25 °C. Then, the seeds were transferred to plates with two sheets of filter paper, soaked in 2 mL of water, and were allowed to germinate under continuous light for 24 h at 25 °C. The percentage of seeds with an emerged radicle was calculated. All tests were conducted thrice. All the IC_50_ values were calculated by IBM SPSS Statistics.

#### 3.3.3. Rice Seedling Growth

Seeds of rice (indica) were sterilized with 70% (*v*/*v*) ethanol for 5 min and washed with distilled water. The sterilized seeds were soaked in water to germinate for three days at 25 °C. Then, the well germinated seeds were selected to place in a glass tube containing 2 mL of a test solution and grown with the tube sealed with a plastic cap under continuous light at 30 °C. The length of the second leaf sheath was measured when the seedlings were seven days old. All of the tests were conducted thrice. All of the IC_50_ values were calculated by IBM SPSS Statistics.

## 4. Conclusions

Collectively, some solid findings were established by the discussion of structure-activity relationship. Introducing of any groups in the benzene ring of *iso*-PhABA was not preferential for bioactivity, but replacing of the benzene ring of *iso*-PhABA to a heterocycle ring can mostly keep the bioactivity, i.e., the IC_50_ values of *iso*-PyABA **5a**, *iso*-FrABA **6** were close to those of control agents **4a** and **1**. Furthermore, the bioactivity of substituted-*iso*-aryl-ABA decreased with an increased size of group in the aryl ring. Also, a double-bond in the 4,5-position of the side chain of *iso*-PhABA analogs was essential for the bioactivity. For the preference of the different bioassays, some new compounds displayed different preference to physiological process comparing with each other and control agents. For example, substituted-*iso*-PhABA **4b**–**4d** observed highest IC_50_ values for the rice seedlings elongation, which was the most efficient assay for the control agents. These results indicated that these analogs might have selectivity and preference to different ABA receptors. As is well known, ABA receptors of plants are multiple gene family [35], and remain unclear for their functions. A selective agonist or antagonist can be used as chemical probe for the discovery of this subject [36]. Thus, those analogs with different preference to bioassays comparing with *iso*-PhABA and ABA might be potential selective agonist and antagonist for ABA receptors, which behave with a different selectivity for the agonist/antagonist-receptor interaction when compared to *iso*-PhABA and ABA. Therefore, these finding made a good opportunity to screening more bicyclic ABA analogs for many important applications, including pointing out the direction for structural modification, practical application on the plant protection and probing the responding of different ABA receptors.

## Figures and Tables

**Table 1:** Inhibitory bioactivities of title compounds.

Compound Name	IC_50_ (μM) ^a^ of Bioassays		
	*Arabidopsis* Seed Germination	Lettuce Seed Germination	Rice Seedlings Elongation
(+)-ABA, **1**	0.39 ± 0.12	0.93 ± 0.06	0.12 ± 0.02
(±)-*iso*-PhABA, **4a**	0.48 ± 0.04	0.65 ± 0.01	0.20 ± 0.03
7′-OMe-*iso*-PhABA, **4b**	2.17 ± 0.52	1.99 ± 0.25	4.71 ± 0.74
6′-OMe-*iso*-PhABA, **4c**	2.11 ± 0.31	3.03 ± 0.28	5.96 ± 0.36
6′-Br-*iso*-PhABA, **4d**	2.81 ± 0.26	3.42 ± 0.46	3.61 ± 0.05
*iso*-PyABA, **5a**	0.57 ± 0.10	1.83 ± 0.35	0.68 ± 0.23
2′-Me-*iso*-PyABA, **5b**	0.91 ± 0.19	9.28 ± 0.62	1.99 ± 0.27
2′-Pr-*iso*-PyABA, **5c**	4.48 ± 0.29	8.22 ± 0.09	>10 ^b^
2′-Ph-*iso*-PyABA, **5d**	>10 ^b^	14.02 ± 3.77	>10 ^b^
*iso*-FrABA, **6**	0.56 ± 0.06	2.77 ± 0.15	0.63 ± 0.14
acetylenic *iso*-PhABA, **24a**	1.38 ± 0.23	7.44 ± 0.53	4.48 ± 0.52
acetylenic 7′-OMe-*iso*-PhABA, **24b**	>10 ^b^	6.50 ± 0.60	5.75 ± 0.28
acetylenic 6′-OMe-*iso*-PhABA, **24c**	7.87 ± 0.65	8.23 ± 0.44	4.41 ± 0.23
acetylenic 6′-Br-*iso*-PhABA, **24d**	6.51 ± 0.51	8.90 ± 0.62	>10 ^b^
acetylenic *iso*-PyABA, **25a**	7.70 ± 0.18	19.69 ± 0.85	9.80 ± 0.50
acetylenic 2′-Me-*iso*-PyABA, **25b**	3.03 ± 0.13	5.97 ± 0.63	8.94 ± 0.66
acetylenic 2′-Ph-*iso*-PyABA, **25d**	>10 ^b^	23.25 ± 5.07	>10 ^b^
acetylenic *iso*-FrABA, **26**	3.45 ± 0.50	8.08 ± 0.02	4.32 ± 0.28

^a^ Required concentrations to inhibit the germinations or seedling elongation by 50%. ^b^ ‘>10’ means that the calculated IC_50_ values are greater than the highest tested concentration, i.e., 10 μM.
